# The impact of the COVID 19 pandemic on the mental health of employees at the Dobrota Kotor Special Hospital for Psychiatry

**DOI:** 10.1192/j.eurpsy.2023.1694

**Published:** 2023-07-19

**Authors:** N. Matkovic, A. Macic, K. Tomcuk, Z. Stevovic, M. Cuckovic, M. Zarkovic, V. Babovic

**Affiliations:** 1 Mental Health - Social Worker; 2 ZU Specijalna Bolnica za Psihijatriju Dobrota, Kotor; 3 Bolnica Risan, Kotor, Kotor, Montenegro

## Abstract

**Introduction:**

The impact of the pandemic on mental health is already evident. In the Special Hospital for Psychiatry Dobrota, Kotor, the number of employees who suffer from anxiety and depression has increased, but the intensity of these disorders has increased in people who were already in some phase of this condition.

**Objectives:**

Besides the influence of Covid 19 pandemic on the phsical health, the pandemic has left a much deeper impact on the mental health of society, especially on the employees of Health Institutions. There is an evident increase of anxiety and depression as well as burn-out syndrome among employees. The purpose of this paper is to investigate the impact of COVID 19 on the mental health of employees at the psychiatric hospital Dobrota, Kotor.

**Methods:**

Data were collected through interviews, anxiety and depression scales from each employee individually. The hospital employs 189 workers, 55 men, 134 women. 42% tested positive once, 12% tested twice or more than once. Of the total number of infected, 86% were women, and 14% were men.

**Results:**

- Our study showed a significant association between the Covid pandemic and mental disorders such as anxiety and depression. These mental disorders were more common in female than in male employees of the Hospital.

**Conclusions:**

In general, the most common feelings are a strong sense of anxiety followed by intense fear and thoughts, tension, excessive caution, increased irritability, resentment, anger and aggression, emotional flatness, anger, sadness and burn out. Depression symptoms that were noticeable were a mood swings, lethargy, a feeling of hopelessness, aimlessness. The anxiety, on the other hand, was determined through various fears, whether a person will find or lose a job, whether he will get sick or not.

**Disclosure of Interest:**

None Declared

